# Silencing of mutant p53 by siRNA induces cell cycle arrest and apoptosis in human bladder cancer cells

**DOI:** 10.1186/1477-7819-11-22

**Published:** 2013-01-28

**Authors:** Hai-Bin Zhu, Kai Yang, Yan-Qi Xie, Yi-Wei Lin, Qi-Qi Mao, Li-Ping Xie

**Affiliations:** 1 Department of Urology, The First Affiliated Hospital, School of Medicine, Zhejiang University, Qingchun Road 79, Hangzhou, Zhejiang Province, 310003, China; 2 Department of Gynecology, The First Affiliated Hospital, School of Medicine, Zhejiang University, Qingchun Road 79, Hangzhou, Zhejiang Province, 310003, China

**Keywords:** Mutant p53, RNA interference, Bladder cancer, Cisplatin, Apoptosis

## Abstract

**Background:**

p53 is the most frequently mutated tumor-suppressor gene in human cancers. It has been reported that mutations in p53 result not only in the loss of its ability as a tumor suppressor, but also in the gain of novel cancer-related functions that contribute to oncogenesis. The present study evaluated the potential of silencing of mutant p53 by small interfering RNA in the treatment of bladder cancer cells *in vitro*.

**Methods:**

We used the 3-(4,5-dimethylthiazol-2-yl)-2,5-diphenyltetrazolium bromide (MTT) assay to assess cell viability and flow cytometry to detect cell cycle alterations and apoptosis. The related molecular mechanisms were assessed by western blotting. We also used the MTT assay and flow cytometry to investigate if silencing of mutant p53 by knockdown with small interfering (si)RNA would change the sensitivity to cisplatin treatment.

**Results:**

Using 5637 and T24 human bladder cancer cell lines characterized by mutations in p53, we found that silencing of the mutant p53 by RNA interference induced evident inhibition of cell proliferation and viability, which was related to the induction of G2 phase cell cycle arrest and apoptosis. Moreover, our study also showed that the p53-targeting siRNA cooperated with cisplatin in the inhibition of bladder cancer cells.

**Conclusions:**

These findings suggest that RNA interference targeting mutant p53 may be a promising therapeutic strategy for the treatment of bladder cancer.

## Background

Bladder cancer is the fourth most common cancer in men, with an estimated 73,510 new cases of and 14,880 deaths from bladder cancer in the USA In 2012 [1]. Although chemotherapy has revolutionized the treatment of advanced tumors [2,3], cisplatin-containing combination chemotherapy for metastatic disease achieves only a median survival of up to 14 months [4], and the associated side-effects induced by its lack of specificity for tumor cells remain a challenging problem. Therefore, novel therapeutic strategies for the treatment of advanced bladder cancer are urgently required.

The well-known tumor suppressor p53 (encoded by the human gene *TP53*), which functions primarily as a transcription factor, plays a vital role in protecting cells from a variety of cellular stresses, including DNA damage and oncogene activation. These stresses trigger the accumulation of p53 protein and activate its transcriptional activity, which prevents cell transformation by inducing cell cycle arrest, senescence, apoptosis, DNA repair, or autophagy [5,6]. *TP53* mutations occur in approximately half of all human cancers, and the majority abrogates the sequence-specific DNA-binding activity of the p53 protein, which constitutes a corner-stone in tumorigenesis [7-9]. Moreover, these mutations usually exert cancer-promoting effects, not only by dominant-negative inactivation of the remaining wild-type allele [10,11], but also through authentic oncogenic gain-of-function activities, which include a wide range of newly acquired oncogenic properties that are not found in the wild-type p53, such as increased genomic instability and cell proliferation, augmented invasion and metastasis, and drug resistance and inhibition of apoptosis [12], underlying the evidence for the association of *TP53* mutations with poor clinical outcome in a variety of cancer types. In support of the gain-of-function hypothesis, stable or conditional knockdown of endogenous p53 mutants in various human cancer cell lines were shown to reduce their proliferation rate and chemoresistance *in vitro*, and their ability to form tumors in nude mice [13,14].

Alterations in the p53 gene and its pathways are usually implicated in muscle-invasive bladder cancer (T2-T4) and advanced stages, and are associated with augmented angiogenesis, invasiveness, metastasis, recurrence, and consequent poor prognosis [15,16]. However, there have been conflicting findings on whether p53 mutations confer increased responsiveness or resistance to cisplatin-based systemic chemotherapy in bladder cancer [17]. In this study, we chose as models two bladder cancer cell lines, T24 and 5637, which both have p53 mutations. The T24 cell line was found to contain a p53 mutation with an in-frame deletion of tyrosine 126 [18], while the 5637 cells carried the p53R280T mutant [19]. Our study showed that knockdown of these p53 mutants by small interfering (si)RNA not only induced cell cycle arrest and cell apoptosis in T24 and 5637 bladder cancer cell lines, but also cooperated with cisplatin in the inhibition of these cancer cells, suggesting that siRNAs may serve as alternative agents for the treatment of bladder cancer by targeting mutant p53.

## Methods

### Reagents

The p53-targeting siRNA used was siP53 (Santa Cruz Biotechnology Inc., Santa Cruz, CA, USA). The control double-stranded small RNA siCon (Table 1) was designed to lack significant homology to all known human sequences and chemically synthesized (GeneChem; Shanghai, China). We used primary immunoblotting antibodies against β-actin, phospho-cyclin-dependent kinase (CDK)1, phospho-CDK2, cyclin A, cyclin B1, cyclin D1, and cyclin E (Cell Signaling Technologies, Beverly, MA, USA), and antibodies against poly(ADP-ribose) polymerase (PARP), CDK1, CDK2, CDK4, caspase-3, caspase-9, and p53 (Santa Cruz Biotechnology Inc., Santa Cruz, CA, USA). This study has been approved by the ethics committee of Zhejiang University and performed according to our institutional guidelines.

**Table 1:** Sequences of RNA and primers used in this study

**Name**	**Direction**	**Sequence 5**^**′**^**→3**^**′**^
siCon	Sense	ACUACUGAGUGACAGUAGA[dT][dT]
	Anti-sense	UCUACUGUCACUCAGUAGU[dT][dT]
P53	Forward	TGCGTGTGGAGTATTTGGATG
	Reverse	TGGTACAGTCAGAGCCAACCTC
GAPDH	Forward	AAGGTGAAGGTCGGAGTCA
	Reverse	GGAAGATGGTGATGGGATTT

### Cell culture and transfection

The human bladder cancer cell lines 5637 and T24 (Shanghai Institute of Cell Biology, Chinese Academy of Science, Shanghai, China) were cultured in RPMI 1640 medium supplemented with 10% heat-inactivated fetal bovine serum, 100 U/ml penicillin, and 100 mg/l streptomycin in a humidified atmosphere containing 5% CO_2_ maintained at 37°C. The day before transfection, cells were plated in growth medium without antibiotics at a density of 30–40%. Transfections of double-stranded (ds)RNA were carried out using transfection reagent (Lipofectamine 2000; Invitrogen, Carlsbad, CA, USA), in accordance with the manufacturer’s protocol. Mock groups were treated with the transfection reagent only. All cell groups were incubated for 24 or 48 hours. Cell images were taken using a phase-contrast microscope at ×100 magnification (Olympus, Japan).

### Real-time quantitative PCR

Total RNA was extracted (TRIzol reagent; Invitrogen, Carlsbad, CA, USA) from the three groups of cells transfected for 48 hours (mock group, 50 nmol/l siCon, and 50 nmol/l siP53) and reverse transcribed using oligo(dT) primers and Moloney murine leukemia virus (M-MLV) reverse transcriptase (Promega, Madison, WI, USA). The resulting cDNA was amplified in a real-time PCR system (ABI Prism 7500; Applied Biosystems, CA, USA) using a commercial reagent (SYBR Premix Ex Taq™ Takara, Dalian, China). Values are expressed as fold difference compared with the mock group. Primer sequences for p53 and GADPH are shown in Table 1.

### Western blotting analysis

Briefly, cells were harvested at 48 hours after dsRNA treatment as described above, washed, and lysed with lysis buffer. Protein concentration in the resulting lysate was determined using the bicinchoninic acid protein assay kit (Pierce Biotechnology Inc., Rockford, IL, USA). Appropriate amounts of protein (30–50 μg) were resolved by electrophoresis in 8 to 12% Tris-glycine polyacrylamide gels and transferred to nitrocellulose membranes. Membranes were blocked and then incubated overnight with the appropriate primary antibody at dilutions specified by the manufacturer. They were next washed three times in 15 ml Tris-buffered saline with Triton (TBS-T) and incubated with the corresponding horseradish peroxidase (HRP)-conjugated secondary antibody at 1:1000 dilution in TBS-T for 1 hour. The membranes were washed three times for 5 minutes each with 15 ml TBS-T, then the bound secondary antibody was detected using an enhanced chemiluminescence (ECL) system (Pierce Biotechnology Inc.).

### Cell growth/viability assay

Proliferation of cells was determined by the (3-(4,5-dimethylthiazol-2-yl)-2,5-diphenyltetrazolium bromide (MTT) assay. Approximately 3000 to 8000 (depending on how long they would be cultured) cells were plated in each well of a 96-well plate. After overnight incubation, the cells were treated with the appropriate dsRNA (mock, 50 nmol/l siCon, or 5–100 nmol/l siP53) for 24, 48, or 72 hours, or with the dsRNA (50 nmol/l siCon or siP53) for 24 hours, followed by incubation for 48 hours with or without 1 μg/ml cisplatin. At the various times after treatment, the medium was removed and MTT (20 μl of a 5 mg/ml solution) was added to each well, and plates were incubated at 37°C for 4 hours. After that, the plates were spun in a centrifuge, and the purple-colored formazan precipitate in each well was dissolved in 150 μl of dimethyl sulfoxide. Absorbance was measured at 490 nm in an absorbance reader (MRX II ; DYNEX Technologies, Chantilly, VA, USA). The reduction in viability of each group was expressed as a percentage of the mock or siCon cells, which were considered to be 100% viable.

### Cell cycle analysis by flow cytometry

Cell cycle analysis was performed using a commercial kit (Coulter DNA Prep™ Reagents Kit; Beckman Coulter, Fullerton, CA, USA). Cells were plated in six-well plates and incubated overnight before treatment (mock, 50 nmol/l siCon and 50 nmol/l siP53 for 48 hours or 50 nmol/l siCon/siP53 for 24 hours with cisplatin or not for 48 hours-). Following treatment, cells were harvested, then washed twice with pre-chilled PBS and resuspended in 100 μl PBS at a concentration of 1×10^6^ cells/ml. Each cell sample was mixed with 100 μl DNA Prep LPR (contained in Coulter DNA Prep™ Reagents Kit), gently mixed by vortex and incubated in the dark at room temperature (25°C) for 20 minutes. Then each was mixed with 1 ml of stain (DNA Prep Stain; contained in Coulter DNA Prep™ Reagents Kit), gently mixed by vortex and again incubated in the dark at room temperature (25°C) for 20 minutes. Finally, cell cycle analysis was performed within 1 hour using flow cytometry (Beckman Coulter FC500 Flow Cytometry System with CXP Software; Beckman Coulter, Fullerton, CA, USA), and the raw data was analyzed by Multicycle for Windows (Beckman Coulter).

### Detection of apoptotic cells by flow cytometry

A quantitative assessment of apoptosis was made by determining the percentage of cells with highly condensed or fragmented nuclei. Cells were harvested at 48 hours after dsRNA treatment (mock, 50 nmol/l siCon, or 50 nmol/l siP53) as described above, washed twice with pre-chilled PBS, and resuspended in 100 μl 1 × binding buffer at a concentration of 1 × 10^6^ cells/ml. Double staining with fluorescein isothiscyanate (FITC)-conjugated annexin V and propidium iodide (PI) was performed (Annexin V-FITC Apoptosis Detection Kit; BD Biosciences, San Jose, CA, USA) in accordance with the manufacturer’s protocol. Cell apoptosis analysis was performed within 1 hour by flow cytometry as described above.

### Statistical analysis

All values are expressed as means ± SD. Statistical significance was compared between treatment groups and controls using Student’s *t* test. *P* < 0.05 was considered significant.

## Results

### Silencing of p53 mutants in bladder cancer cells

**Figure 1:**
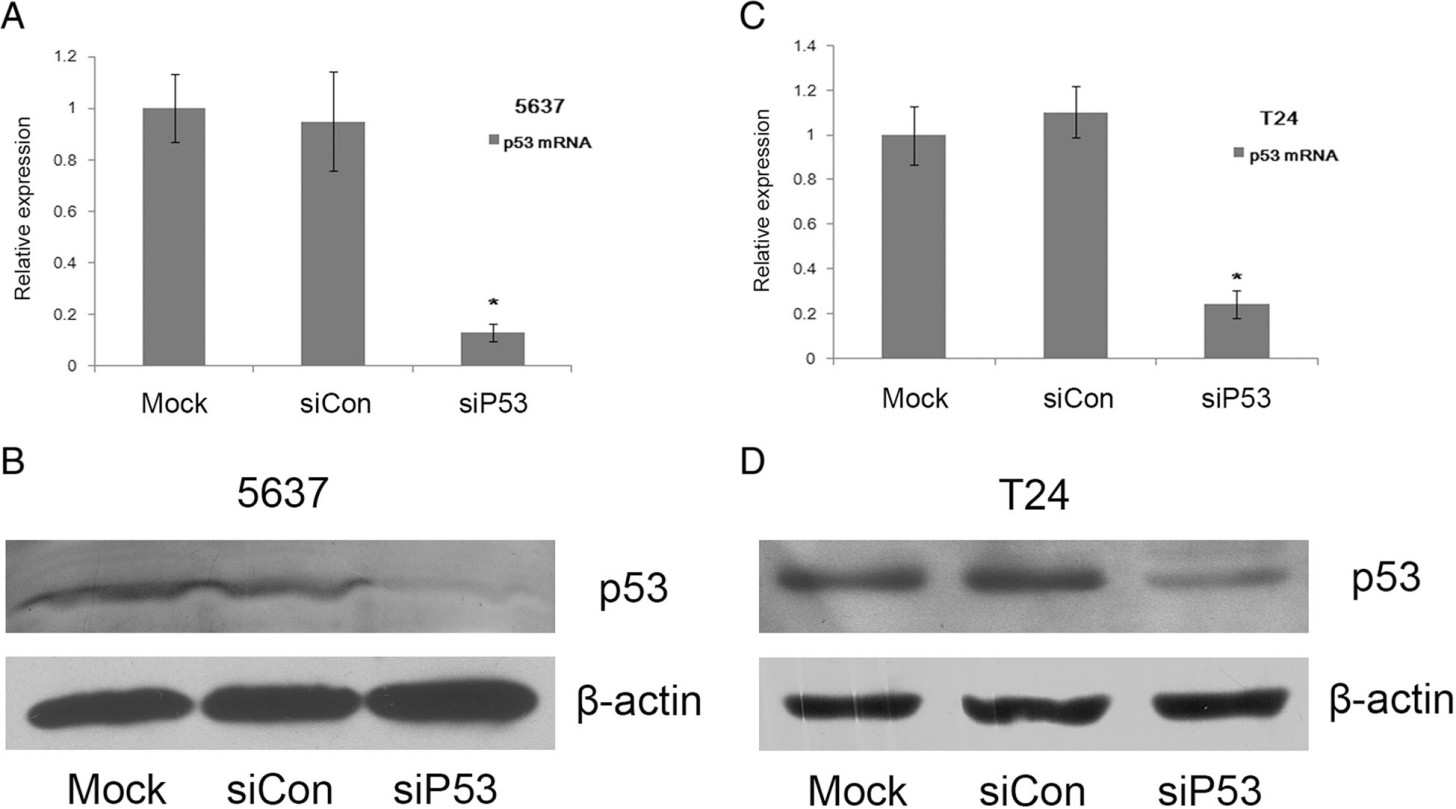
**A p53-targeting small interfering (si)RNA (siP53) reduced p53 expression in T24 and 5637 human bladder cancer cells.** p53 mRNA expression in **(A)** 5637 and **(C)** T24 cells transfected by siP53 or siCon were assessed by real-time quantitative PCR. The results were normalized to GAPDH and are presented as the mean ± SD of three independent experiments. * *P* < 0.05 versus mock and small interfering control (siCon) groups. The p53 protein levels in **(B)** 5637 and **(D)** T24 cells were assessed by western blotting analysis. β-actin levels were also assessed and served as a loading control. A representative blot is shown from three independent experiments with identical results.

First, we checked the effects of the p53-targeting siRNA (siP53) on the expression of endogenous mutant p53 in 5637 and T24 human bladder cancer cell lines, which had been transfected with 50 nmol/l siP53 or a control dsRNA (siCon). At 48 hours after transfection, expression of p53 mRNA and protein was assessed by real-time quantitative PCR and western blotting respectively. Compared with the mock and siCon transfections, siP53 caused a reduction of more than 70% reduction in expression of the mutant p53 (Figure 1).

### Knockdown of mutant p53 inhibits the growth and viability of 5637 and T24 cells

**Figure 2:**
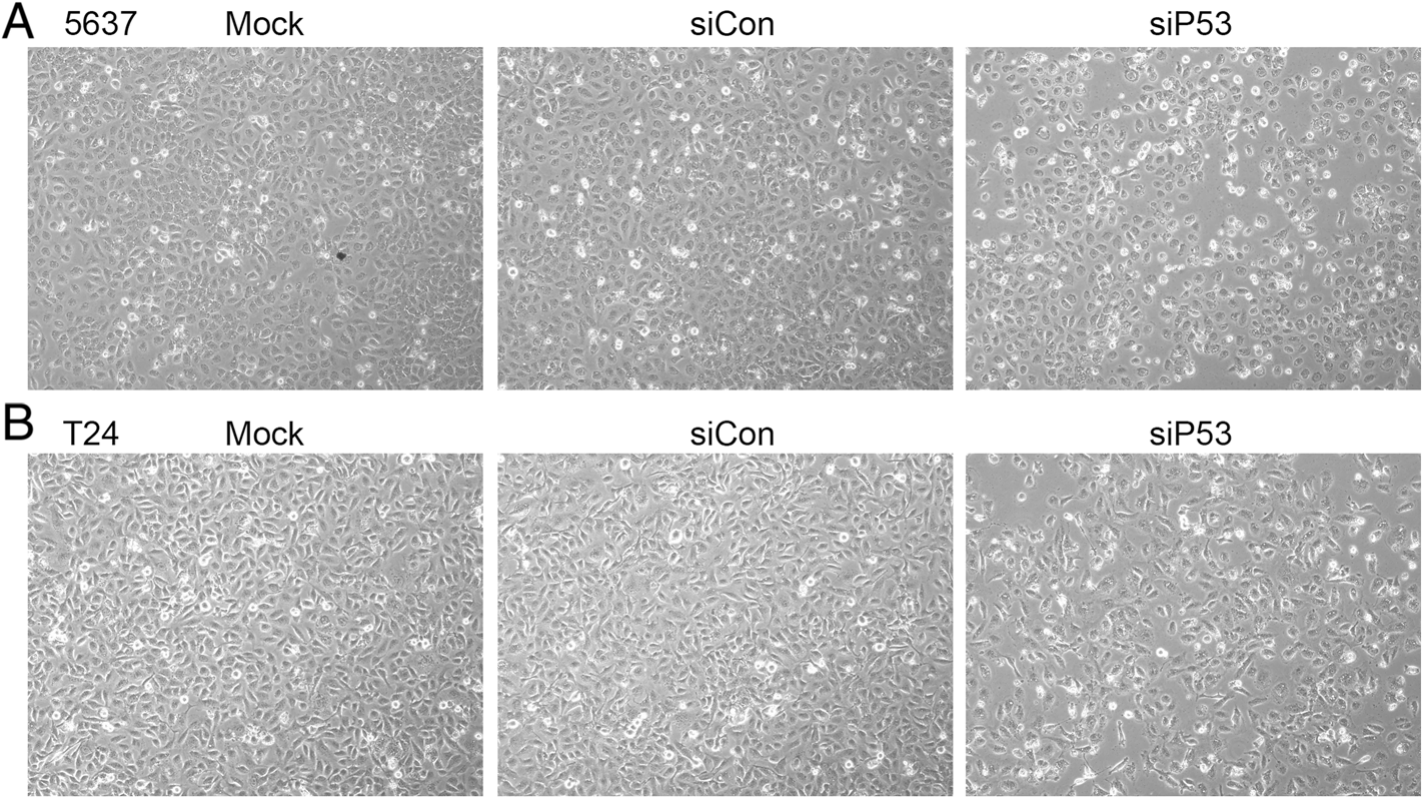
**The p53-targeting small interfering (si)RNA (siP53) inhibited the growth of cells. (A)** 5637 and **(B)** T24 cells were transfected with 50 nmol/l siP53, 50 nmol/l siCon or mock RNA. Cell images were taken at 48 hours after transfection at 100× magnification. The siP53-transfected cells were less dense and had more dead cells in the culture than the cells in the siCon and mock groups.

We next investigated the effects of knockdown of mutant p53 on bladder cancer cells. The two dsRNAs, siP53 and siCon, were respectively transfected into 5637 and T24 cells at a concentration of 50 nmol/l. Phase-contrast images of cells from the same fields were taken 48 hours after transfection. Morphologically, the mock and siCon transfected cells maintained healthy growth, whereas the cells transfected with siP53 lost viability, and there was an evident decrease in cell number (Figure 2).

**Figure 3:**
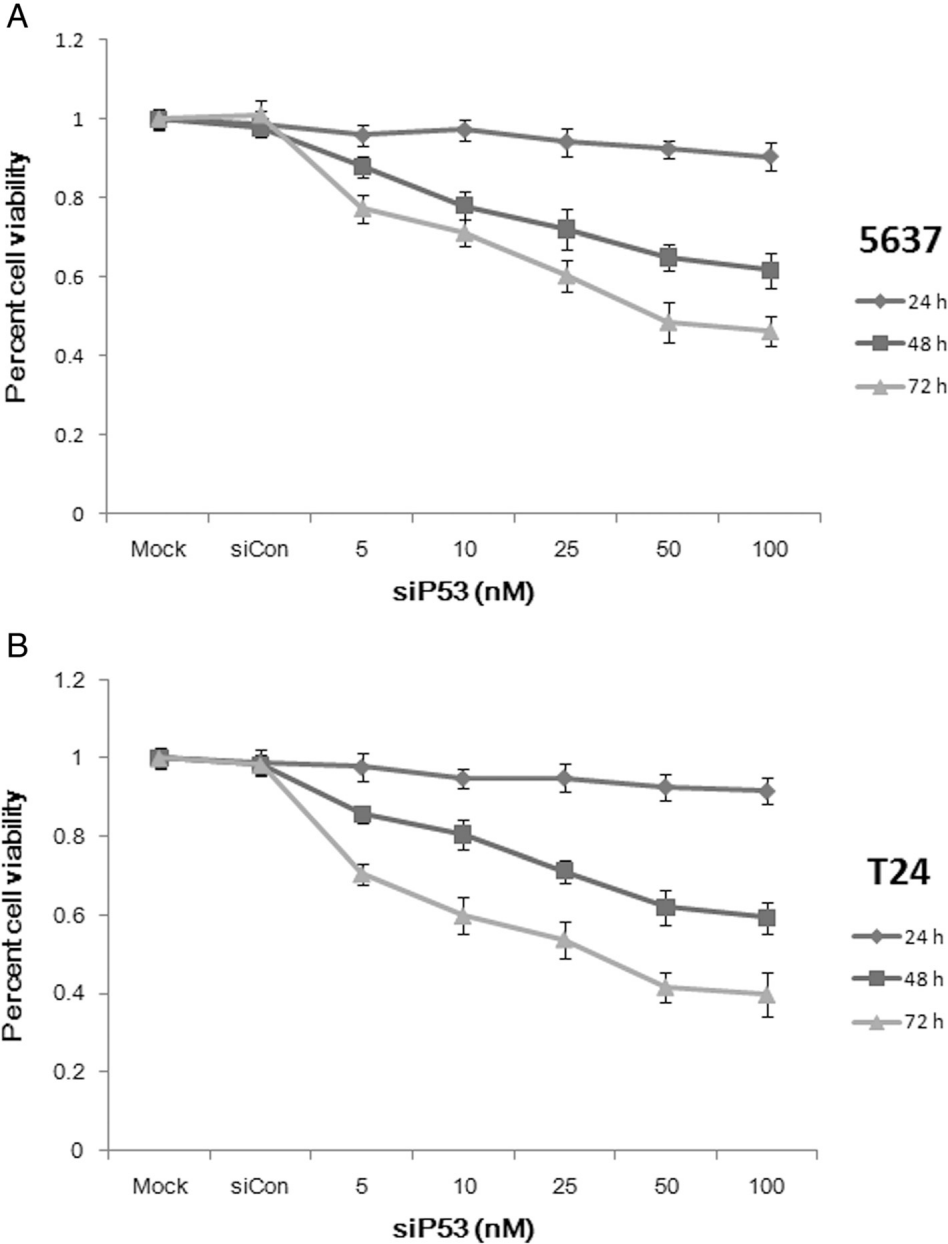
**The small interfering (si)P53 inhibited the viability of cells in a dose-dependent and time-dependent manner, as assessed by the (3-(4,5-dimethylthiazol-2-yl)-2,5-diphenyltetrazolium bromide (MTT) assay.** For both **(A)** 5637 and **(B)** T24 cells, reduced cell viability was seen after siP53 treatment (5 to 100 nmol/l) at 24, 48, and 72 hours. Data are presented as means ± SD (n = 8).

Following this, the effects of siP53 at varying concentrations and times (24 to 72 hours) on the proliferation and viability of 5637 and T24 cells were determined by the MTT assay. Inhibition of cell growth and viability by siP53 was dependent on dose and time (Figure 3). Reduction in 5637 cell viability with siP53 treatment at concentrations of 5 to 100 nmol/l after 72 hours ranged from 22.7% to 53.8%, whereas reduction of cell viability in T24 cells ranged from 29.4% to 60.3% (Figure 3).

### Silencing of mutant p53 induces G2-phase cell cycle arrest in bladder cancer cells

**Figure 4:**
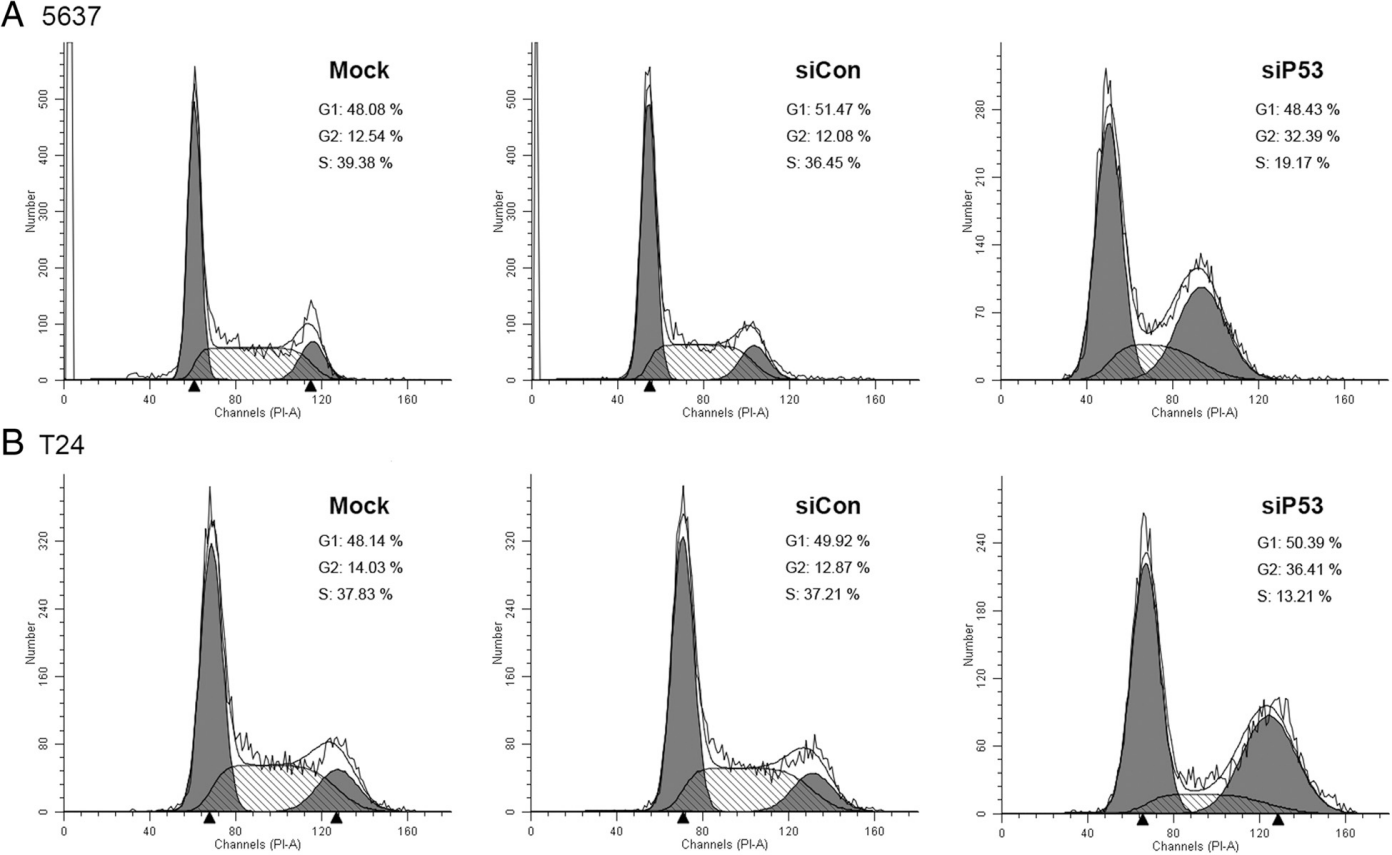
**Treatment with 50 nmol/l small interfering (si)P53 induced G2-phase cell cycle arrest in human bladder cancer cells detected by flow cytometry. (A)** 5637 and **(B)** T24 cells. The sub-G0/G1 cells were not included in the calculations. A representative image is shown from three independent experiments with identical results, and these data are the average results of three independent experiments.

In addition to their ability to interfere with wild-type p53-mediated cell cycle arrest, many mutant p53 proteins can actively promote cell growth by affecting various proliferation-related genes [20,21]. Therefore, we assessed whether siP53-induced inhibition of growth and viability is mediated via alterations in cell cycle regulation. The supernatant including suspended cells was removed and adherent live cells were harvested after 48 hours of treatment. G2 phase arrest was seen in cells treated with 50 nmol/l siP53 (Figure 4). The G2-phase population of the siP53-treated 5637 cells was 32.39% at 48 hours after treatment, and increased by about 20% compared with controls, whereas the percentage of G2-phase T24 cells increased about 23% after 48 hours of treatment. The increase in cell population in the G2 phase was found to be associated with a concomitant significant decrease in the S-phase population.

**Figure 5:**
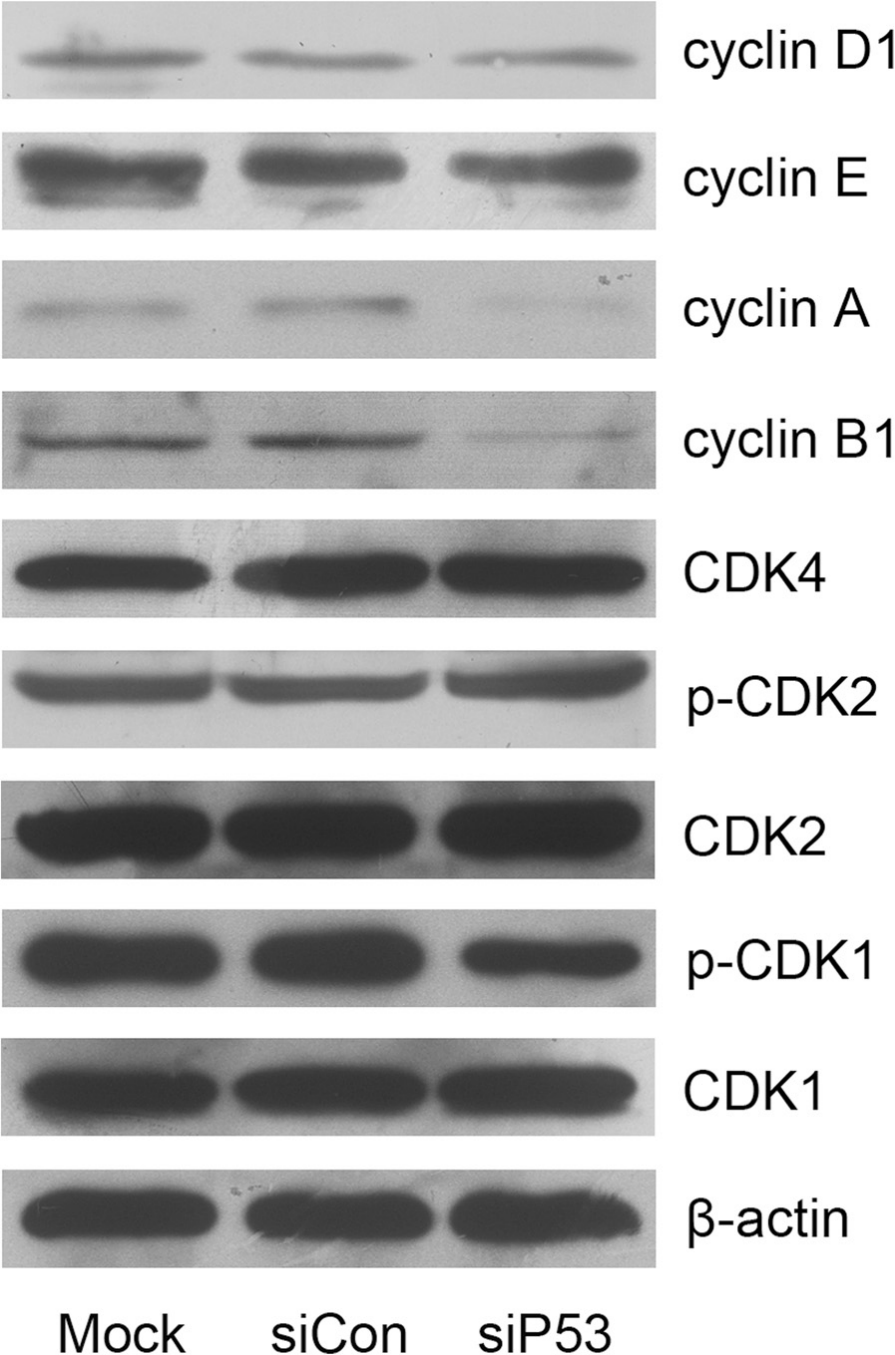
**The effects of small interfering (si)P53 on cell cycle-related proteins in 5637 cells.** Cell lysates were analyzed by immunoblotting. A representative blot is shown from three independent experiments with identical results.

Next, we examined the expression of several cell cycle-related proteins. Interference with the cell cycle in 5637 cells was associated with decreased expression of cyclin B1 and cyclin A, and reduced phosphorylation of CDK1, but no alteration in the expression of other cyclins and CDKs (Figure 5). The results of T24 cells are similar (data not sown).

### Silencing of mutant p53 induces cell apoptosis in bladder cancer cells

**Figure 6:**
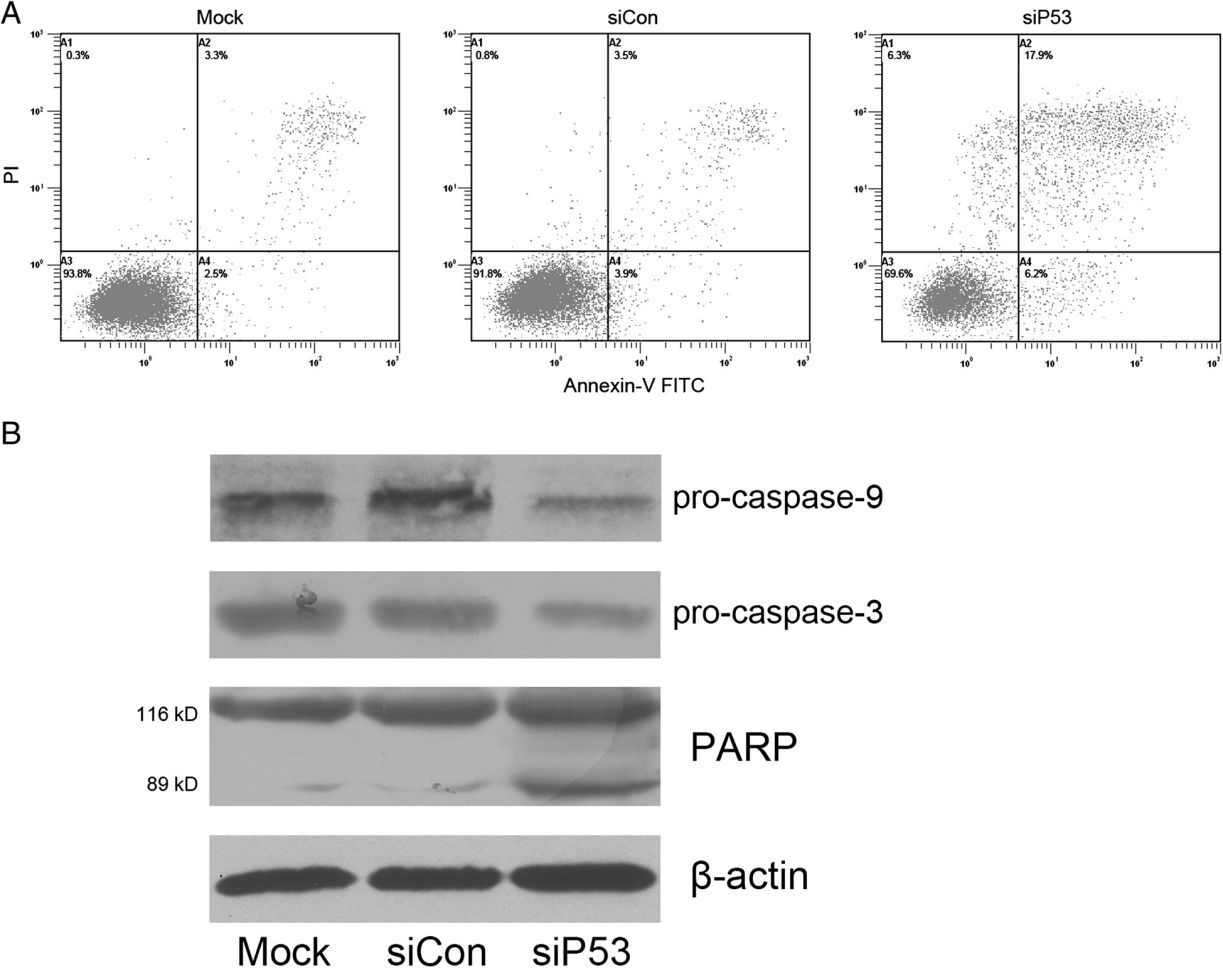
**Small interfering (si)P53-induced apoptosis in 5637 human bladder cancer cells. (A)** siP53 treatment resulted in apoptosis in 5637 cells detected by flow cytometry using a double-staining method with fluorescein thiocyanate-conjugated annexin V and propidium iodide. **(B)** siP53 treatment activated caspase-3, caspase-9, and poly(ADP-ribose) polymerase (PARP) in 5637 cells. Representative images and blots from three independent experiments with identical results are shown, and these data are the average results of three independent experiments.

We next investigated the relationship between siP53-mediated loss of cell viability and apoptosis by flow-cytometry analysis of 5637 cells labeled with PI and annexin V. We found that after 72 hours of treatment, siP53 caused evident apoptosis in 5637 cells. The number of early apoptotic cells (LR quadrant) increased to 6.2% in 5637 cells, and the number of late apoptotic cells (UR quadrant) increased to 17.9% (Figure 6A). These data also showed that siP53 treatment resulted in 6.3% cell necrosis, which might be a secondary event in the apoptotic process. Similar results were also seen in T24 cells (data not shown).

Accordingly, activation of caspase 9 and caspase 3 and cleavage of PARP, which represent one of the final steps of the proteolytic caspase cascade and reliably indicate ongoing apoptosis, were present in siP53-treated 5637 cells (Figure 6B).

### Silencing of mutant p53 cooperates with cisplatin in producing inhibition of bladder cancer cells

Cisplatin is the main chemotherapy drug for advanced bladder cancer. As explained in the Introduction, there have been conflicting findings as to whether p53 mutations confer increased responsiveness or increased resistance to cisplatin-based systemic chemotherapy in bladder cancer [17]. Thus, we used MTT assay to determine the combined effects of siP53 and cisplatin on 5637 bladder cancer cells.

**Figure 7:**
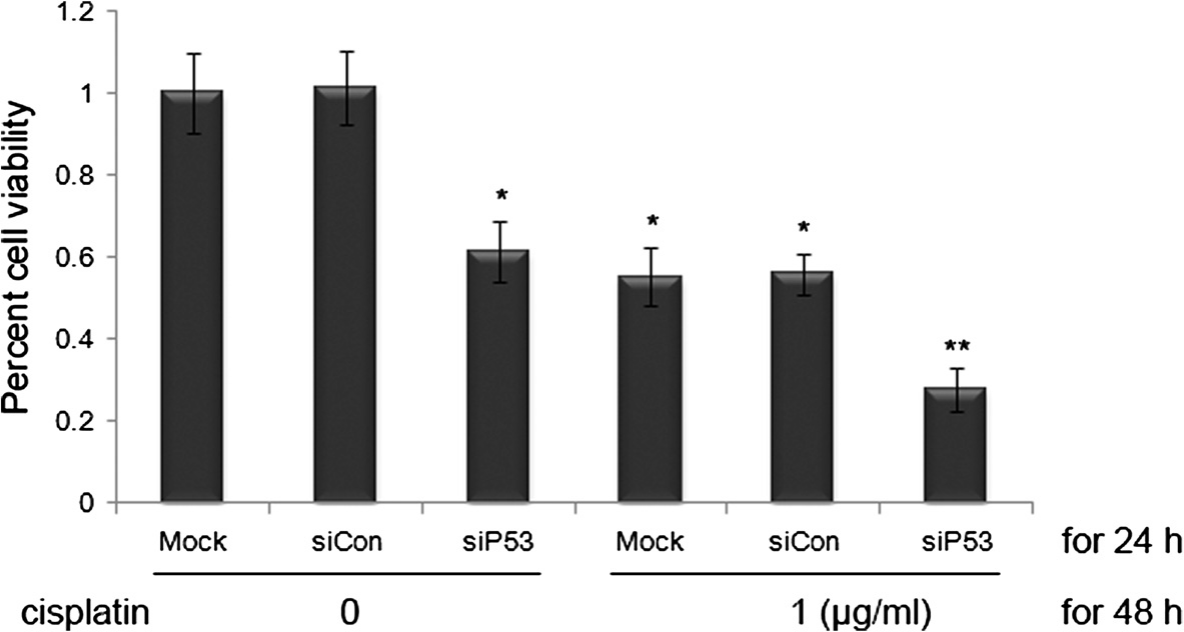
**Silencing of mutant p53 cooperates with cisplatin in inhibiting the viability and growth of 5637 cells assessed by the (3-(4,5-dimethylthiazol-2-yl)-2,5-diphenyltetrazolium bromide (MTT) assay.** These data are presented as means ± SD (n = 8). * *P* < 0.05 versus single treatment with the conrol (siCon) or mock DNA. ** *P* < 0.05 versus single treatment with siP53 + cisplatin.

After transfection with mock, siCon, or siP53 siRNAs for 24 hours, cells were sub-divided into two groups, and treated or not with 1 μg/ml cisplatin for another 48 hours. The average reduction in cell viability with siP53 + cispaltin treatment was 72.3% (Figure 7), which was much greater than the reduction obtained with the single treatment of siP53 (38.7%) or cisplatin (44.9%). Similar results were also seen in T24 cells (data not shown).

**Figure 8:**
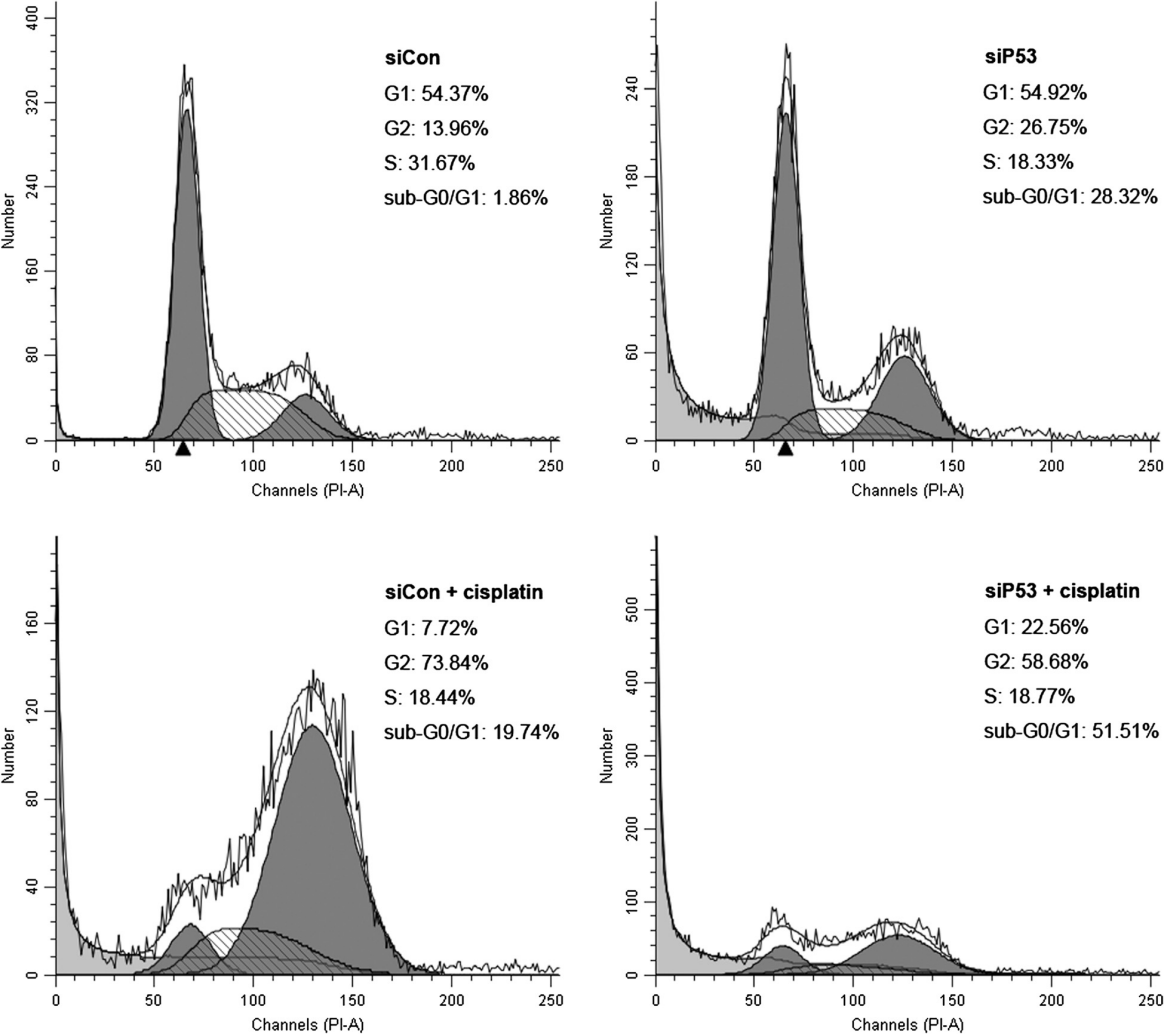
**Silencing of mutant p53 cooperates with cisplatin in inducing apoptosis in 5637 cells assessed by flow cytometry.** The percentage of cells in G1, S, or G2 phase equals the number of cells in each phase divided by the number in all three phases, and the percentage of sub-G0/G1 cells equals the number of sub-G0/G1 cells divided by the number of all cells counted. A representative image is shown from three independent experiments with identical results, and these data are the average results of three independent experiments.

Both the live cells and the supernatant were then harvested to assess the effects of the treatment above by flow cytometry. Compared with the siCon-treated group, G2-phase arrest was seen in both the siP53-treated and cisplatin-treated groups (Figure 8). Moreover, there was a significant increase in the sub-G0/G1 population in these two groups (28.32% and 19.74%, respectively), indicating apoptotic cells. When siP53-transfected cells were subsequently treated with cisplatin for 48 hours, the proportion of sub-G0/G1 cells increased to 51.51%, suggesting that the p53-targeting siRNA can co-operate with cisplatin in the inhibition of bladder cancer cells.

## Discussion

Mutations in the *TP53* gene, encoding the p53 tumor suppressor, are arguably the most common type of gene-specific alterations in human cancer. This emphasizes the pivotal role of p53 as a major mainstay of the body’s built-in anticancer defense mechanisms. As the field of p53 research evolves, it is increasingly evident that many p53 mutants not only lose their own tumor-suppressive functions and acquire dominant-negative activities over the remaining wild-type allele, but also gain new oncogenic properties that are not present in wild-type p53; this phenomenon is termed the ‘gain-of-function hypothesis’. For instance, overexpression of p53 mutants in cultured cells was shown to interfere with apoptosis, enhance proliferation, and increase resistance of the cells to chemotherapy [22-24]. Moreover, transfection of mutant p53 into *TP53*-null cells was shown to enhance their ability to form tumors in mice [25]. Therefore, whereas wild-type p53 is a potent tumor suppressor, cancer-associated p53 mutants possess the attributes of oncogenes, suggesting that knockdown of mutant p53 may restrain or reverse the process of oncogenesis.

Recently, therapeutics based on RNA interference (RNAi) have become powerful and useful methods for the treatment of many diseases, including cancer, because of the high specificity, high efficacy and low toxicity of the RNAi trigger, small dsRNA [26,27]. Stable or conditional knockdown of endogenous mutant p53 by siRNAs in various human cancer cell lines, such as lung, breast, and colon cancer cells, has been reported to reduce their growth rate and chemoresistance *in vitro*, and their ability to form tumors in nude mice [13,14,28]. A recent study also obtained consistent results showing that siRNA targeting mutant p53 could induce cell cycle arrest and apoptosis in human prostate cancer cells [29]. In the current study, we used siRNAs that targeted p53 mutants to transfect human bladder cancer cell lines expressing only p53 mutants endogenously, and found that the transfection resulted in suppressed cell growth and viability via induction of cell cycle arrest and apoptotic cell death.

In numerous cancer types, mutations in *TP53* are strongly associated with high expression levels of the proliferation-associated gene cluster, comprised mainly of the genes that participate in the core processes of the cell cycle [30]. Moreover, several p53 mutants were recently shown to repress wild-type p53 target genes, which encode pivotal cell cycle inhibitors (such as p21 and GADD45α (growth arrest and DNA-damage-inducible 45α)), leading to alteration of cyclins/CDKs and an increased proliferation rate [31]. Whereas overcoming the G2/M checkpoint to initiate mitosis requires cyclin B/CDK1 to be activated, cyclin A seems to be required for both S-phase and M-phase [32]. Cyclin A/CDK2 drives G2-phase cells into mitosis, and is a rate-limiting component required for entry into mitosis [33]. Our analysis of cell cycle-related proteins showed that in the siP53-treated cells, there was significantly decreased expression of both cyclin B1 and cyclin A and reduced phosphorylation of CDK1, supporting the induction of G2 phase arrest detected by flow cytometry. Meanwhile, our study also showed activation of caspase-9 and caspase-3 and proteolytic cleavage of PARP in these cells, which play central roles during cell apoptosis [34,35].

Over the past two decades, cisplatin-based combination chemotherapy regimens, such as CMV (cisplatin, methotrexate, and vinblastin) or M-VAC (methotrexate, vinblastin, doxorubicin, and cisplatin) have been mainly used for patients with advanced bladder cancer [36-39]. However, because of their severe systemic toxicity and the poor overall prognosis of patients, novel therapeutic schemes containing different drug cocktails have been developed, with cisplatin occupying a central position in theseregimen (for example the GC (gemcitabine + cisplatin) regimen) [40]. It is generally accepted that DNA is the preferential and cytotoxic target for cisplatin [41-43]. Cisplatin-mediated damage of genomic DNA causes severe cell cycle perturbation and arrest at certain checkpoints, and in the absence of adequate repair, the affected cells undergo cell apoptosis. There has been controversy about the influence of mutant p53 on the responsiveness to the cisplatin-based systemic chemotherapy in bladder cancer [17]. Several studies have reported that patients with p53-altered bladder cancer benefited from adjuvant chemotherapy [44], whereas wild-type p53 was related to a poor response to systemic cisplatin-based chemotherapy [45], which might be related to the protection of cells from DNA damage by wild-type p53. However, other studies showed that mutations in *TP53* were associated with drug resistance in several malignancies and cell lines [22,46], which might be partially attributable to transcriptional activation of the multi-drug resistance 1 (*MDR1*) gene [47,48] and interference with apoptosis [23] by mutant p53. Therefore, targeting mutant p53 may sensitize the cancer cells to chemotherapy in at least some bladder cancers. In the current study, we found that knockdown of mutant p53 by siRNA in 5637 and T24 bladder cancer cells could co-operate with cisplatin and enhance its anticancer effects additively via increased cell apoptosis.

## Conclusions

Our study indicated that knockdown of mutant p53 by siRNA was able to induce G2-phase cell cycle arrest and apoptosis in 5637 and T24 human bladder cancer cells. Moreover, this strategy cooperated with cisplatin in the inhibition of bladder cancer cells. Our results provide evidence that targeting mutant p53 by RNAi may serve as a promising therapeutic strategy for the treatment of partial advanced bladder cancer bearing p53 mutations. Despite the promise, the principal challenges that remain in achieving the broadest application of RNAi therapeutics are the hurdles of delivery and target validation *in vivo*. Further studies are needed to delineate the exact mechanism and verify *in vivo* effectiveness for clinical use in the future.

## Competing interests

The authors declare that they have no competing interests.

## Authors’ contributions

HBZ, KY and LPX conceived the study concept and participated in its design, data extraction, statistical analysis, manuscript drafting and editing. KY, YQX and YWL performed the experiment. QM participated in design and manuscript drafting. All authors read and approved the final manuscript.
